# Feasibility Domain Construction and Characterization Method for Intelligent Underground Mining Equipment Integrating ORB-SLAM3 and Depth Vision

**DOI:** 10.3390/s26030966

**Published:** 2026-02-02

**Authors:** Siya Sun, Xiaotong Han, Hongwei Ma, Haining Yuan, Sirui Mao, Chuanwei Wang, Kexiang Ma, Yifeng Guo, Hao Su

**Affiliations:** 1 College of Electrical and Control Engineering, Xi’an University of Science and Technology, Xi’an 710054, China; sunsiya412@xust.edu.cn (S.S.); tamela@foxmail.com (H.Y.); 24206204126@stu.xust.edu.cn (S.M.); 2 Shaanxi Key Laboratory of Mine Electromechanical Equipment Intelligent Detection and Control, Xi’an 710054, China; mahw@xust.edu.cn (H.M.); wangchuanwei228@xust.edu.cn (C.W.); 22105016005@stu.xust.edu.cn (K.M.); guoyifeng000721@163.com (Y.G.); suh@stu.xust.edu.cn (H.S.); 3 College of Mechanical Engineering, Xi’an University of Science and Technology, Xi’an 710054, China

**Keywords:** underground coal mine, simultaneous localization and mapping, feasible domain construction

## Abstract

To address the limited environmental perception capability and the difficulty of achieving consistent and efficient representation of the workspace feasible domain caused by high dust concentration, uneven illumination, and enclosed spaces in underground coal mines, this paper proposes a digital spatial construction and representation method for underground environments by integrating RGB-D depth vision with ORB-SLAM3. First, a ChArUco calibration board with embedded ArUco markers is adopted to perform high-precision calibration of the RGB-D camera, improving the reliability of geometric parameters under weak-texture and non-uniform lighting conditions. On this basis, a “dense–sparse cooperative” OAK-DenseMapper Pro module is further developed; the module improves point-cloud generation using a mathematical projection model, and combines enhanced stereo matching with multi-stage depth filtering to achieve high-quality dense point-cloud reconstruction from RGB-D observations. The dense point cloud is then converted into a probabilistic octree occupancy map, where voxel-wise incremental updates are performed for observed space while unknown regions are retained, enabling a memory-efficient and scalable 3D feasible-space representation. Experiments are conducted in multiple representative coal-mine tunnel scenarios; compared with the original ORB-SLAM3, the number of points in dense mapping increases by approximately 38% on average; in trajectory evaluation on the TUM dataset, the root mean square error, mean error, and median error of the absolute pose error are reduced by 7.7%, 7.1%, and 10%, respectively; after converting the dense point cloud to an octree, the map memory footprint is only about 0.5% of the original point cloud, with a single conversion time of approximately 0.75 s. The experimental results demonstrate that, while ensuring accuracy, the proposed method achieves real-time, efficient, and consistent representation of the 3D feasible domain in complex underground environments, providing a reliable digital spatial foundation for path planning, safe obstacle avoidance, and autonomous operation.

## 1. Introduction

In recent years, with the vigorous advancement of intelligent coal mining in China, national ministries and commissions have successively issued several key support project guidelines for intelligent coal mine transformation in response to major demands within the coal industry [1,2,3,4]. Autonomous environmental perception technology serves as a prerequisite for achieving efficient, reduced-manpower, and intelligent underground mining equipment. Its technical challenge lies in high-precision, real-time environmental mapping and digital representation methods within enclosed underground spaces [5].

Facing complex underground environments characterized by weak textures, high dust levels, and localized intense lighting, the evolution of environmental perception for intelligent equipment and the construction of navigable workspace areas has transitioned from fixed geometric space planning to multi-sensor fusion modeling [6]. Su Guoyong’s team introduced ELAN-DS and the decoupled-head CMCE-Net into YOLOv7-tiny, achieving over 90% detection accuracy even under complex coal mine conditions, demonstrating the reliability of deep vision for underground perception [7]. Beyond visual sensors, Zhou et al. addressed environmental perception challenges in autonomous rubber-tired vehicles underground by combining SLAM with Ultra-Wideband (UWB) Inertial Navigation Systems (INS) for positioning [8]. At the localization and mapping level, Ma Hongwei’s team proposed a LiDAR-IMU tightly coupled SLAM framework. Through front-end filtering and back-end map optimization, this framework effectively suppresses longitudinal drift, reduces cumulative system errors, and achieves higher accuracy, real-time performance, and robustness, ensuring global consistency in tunnel map construction [9]. Thus, the integration of deep vision and SLAM technologies has emerged as a research hotspot for intelligent equipment perception in coal mine work spaces.

Regarding SLAM technology, the ORB-SLAM2 system offers high precision and real-time stability. However, it still suffers from issues such as sparse map construction, large storage requirements, and inability to digitally describe spaces, rendering it unsuitable as a prerequisite for intelligent equipment path planning and higher-level human–machine interaction [10,11,12,13]. Han Yanfeng et al. proposed an improved ORB-SLAM2 algorithm that utilizes depth information to obtain the 3D positions of keyframe point clouds via a pinhole imaging model. Outliers and redundant points are removed through voxel filtering, and the keyframe point cloud poses are stitched and optimized to generate a dense point cloud map [14]. Rosinol et al.’s ORB-SLAM3 achieves dense reconstruction at the geometric level while integrating semantic segmentation results from the feasible region into the pose map optimization framework, thereby producing dense maps that combine geometric accuracy with semantic representation [15]. Zhang D et al. pointed out that traditional ORB-SLAM3 systems inherently follow a feature-point-cloud-dominated SLAM framework rather than performing pixel-level depth fusion for dense reconstruction. Consequently, reconstruction errors are amplified in complex regions of the described object, leading to imprecise 3D structures manifested as weak densification and poor reconstruction accuracy in dense maps [16]. Studies have shown that the environmental perception ability of intelligent equipment is significantly constrained in the complex environment of underground coal mines. Marek et al. found that in situations with high dust concentration or insufficient lighting, the performance of traditional feature-point-based SLAM algorithms deteriorates significantly, making it difficult to meet the requirements of intelligent equipment in underground mines for environmental modeling accuracy and continuity [17]. At the same time, Xianyao et al. demonstrated that in the complex environment of coal mines, improving the robustness of the perception system not only depends on the improvement of the SLAM algorithm itself, but also on the effective modeling and processing of sensor data quality [18].

In response to these issues, this paper proposes a method that utilizes RGB-D depth cameras and an improved ORB-SLAM3 algorithm to construct an octree map. This approach provides a technical foundation for the digital description of the working space’s feasible area by intelligent equipment in coal mines under dusty and uneven lighting conditions.

The main innovations and contributions of this paper are as follows:

1. This work establishes an ORB-SLAM3-based framework for 3D feasible-domain modeling in underground coal mines. By leveraging the high-accuracy pose estimation provided by ORB-SLAM3 and integrating RGB-D depth sensing with dense 3D reconstruction, the proposed system enables coordinated localization and feasible-space representation, providing a systematic solution for digital modeling of complex operational spaces.
2. This paper proposes a dense reconstruction method based on ChArUco calibration and multi-stage depth optimization, aiming to improve the mapping quality in complex environments. By combining high-precision ChArUco calibration with mathematical projection models, stereo matching, and multi-stage depth filtering, it effectively addresses the issues of sparse dense point clouds and missing details under weak texture and low illumination conditions, providing a reliable geometric foundation for the subsequent construction of the feasible region’s voxelization.
3. The dense point cloud is converted into a probabilistic octree occupancy map, where voxel updates are performed only for observed space. We further model the computational complexity of the feasible-space construction process and derive a per-frame time complexity of O(R⋅k⋅d), where R is the number of depth rays, k is the number of voxels traversed per ray, and d is the octree depth. In addition, we show that memory usage grows approximately linearly with the spatial scale. Together, theoretical analysis and experimental results confirm the scalability and real-time capability of the proposed method.

## 2. Three-Dimensional Reconstruction System Based on ORB-SLAM3

ORB-SLAM3, as an efficient multimodal visual SLAM system, extends the ORB-SLAM2 and ORB-SLAM-VI [19] architectures to a multi-map framework, supporting multi-session operation in pure visual, visual-inertial, and inertial-only modes.

Its core functionality is implemented through four cooperating threads: The tracking thread extracts ORB features from input images and matches consecutive frames. It iteratively optimizes the initial camera pose by minimizing re-projection error, establishing associations between feature points and map points to maintain the active map. The local mapping thread inserts keyframe poses and new map points into the active map, employs sliding-window beam-forming for BA optimization of map structure, and combines pre-integration with re-projection error to refine keyframes while pruning redundant data; The loop closure detection thread performs cross-map common view region analysis on filtered keyframes. Upon detecting a loop closure or map fusion requirement, it executes similarity transformation-based closed-loop correction and map merging. An independent thread asynchronously updates maps to ensure real-time performance [20]. The Atlas dynamically manages active maps (currently tracked local maps) and inactive sub-maps (historical sub-maps retained after tracking loss). When closed loops exist between them, they are merged to generate new active maps [21].

## 3. Camera Calibration and Enhanced 3D Reconstruction Methods

Addressing issues such as high error rates and low accuracy when using traditional calibration boards for depth camera selection and calibration in complex enclosed environments, we generate a novel ChArUco calibration board by integrating ArUco QR codes [22] with the original Chessboard calibration board [23]. Calibration using this board provides accurate geometric and distortion parameters for subsequent stereo vision and depth estimation tasks.

### 3.1. Depth Cameras and Calibration Principles

Depth cameras are currently categorized into three types: structured light cameras [24], TOF cameras [25], and stereo depth cameras [26]. This paper employs a stereo depth camera as the sensor.

The principle of stereo depth cameras involves using left and right cameras to capture image position information, estimating the distance from the target to the camera by calculating the disparity between the two images. The RGB-D stereo camera used in this paper is the OAK-D-PRO-POE. The stereo camera baseline of the camera refers to the physical distance between the two cameras (one on the left and one on the right) used for depth calculation, which is approximately 75 mm.

To determine the correspondence between the three-dimensional position information of a point on a spatial object’s surface and its location in the images, it is necessary to establish the camera’s imaging geometric model. The parameters within this geometric model constitute the camera parameters. Through camera calibration, the internal and external parameters of the camera, along with distortion parameter data, can be accurately obtained. This provides an accurate geometric model for image processing and machine vision applications, enabling precise measurement and localization of object positions and shapes.

Traditional camera calibration typically utilizes a Chessboard calibration board, which relies on the detection of continuous chessboard corner points. If parts of the area are obscured or lighting is uneven, corner points may be lost or misdetected. Corner detection relies on pixel gradients at the black-white boundaries of the chessboard pattern, making it susceptible to image blur, distortion, or low resolution [27]. This significantly reduces calibration precision and accuracy in high-precision depth camera calibration. Against this backdrop, a new calibration board is required. In contrast, the ChArUco calibration board offers superior corner localization accuracy and occlusion resistance, but it is more complex to implement.

**Figure 1:**
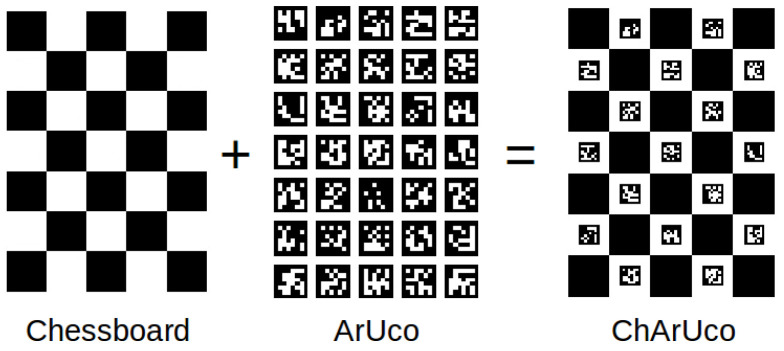
Definition of the ChArUco Calibration Plate.

As shown in Figure 1, the ChArUco calibration board is a hybrid calibration plate integrating traditional chessboard patterns with ArUco markers, specifically designed for high-precision camera calibration and robust detection. It provides sub-pixel-level coordinate accuracy via the chessboard’s corner points for calibrating the camera’s internal parameters (focal length, distortion, etc.). During calibration, when portions of the chessboard are obscured, the ArUco markers use their unique IDs to infer the positions of the hidden corners, enhancing robustness. The sub-pixel accuracy of the ChArUco chessboard corners surpasses that of pure ArUco calibration. By combining the strengths of both chessboard and ArUco markers, the ChArUco calibration board significantly improves calibration reliability and precision in complex environments.

When calibrating with the ChArUco plate, the system first extracts ArUco markers and grid corner points. When grid corners are obscured, surrounding ArUco markers are used to interpolate the grid corners, as illustrated by Equation (1):

(1) $$P_{hidden}=\frac{\sum_{k=1}^{4}w_{k}\cdot P_{aruco_{k}}}{\sum_{k=1}^{4}w_{k}}$$

where $w_{k}$ represents the weighting factor used to estimate occluded corner points through interpolation based on the positions of adjacent ArUco markers. The detected corner points are then processed using the OpenCV [28] library to enhance accuracy to sub-pixel levels, with gradient information employed to optimize corner locations. The underlying principle is illustrated by Equations (2)–(4):

(2) $$\Delta x=\frac{-\left(\sum_{k=1}^{N}I_{y,k}^{2}\right)\left(\sum_{k=1}^{N}I_{x,k}I_{k}\right)+\left(\sum_{k=1}^{N}I_{x,k}I_{y,k}\right)\left(\sum_{k=1}^{N}I_{y,k}I_{k}\right)}{\det(H)}$$

(3) $$\Delta y=\frac{\left(\sum_{k=1}^{N}I_{x,k}I_{y,k}\right)\left(\sum_{k=1}^{N}I_{x,k}I_{k}\right)-\left(\sum_{k=1}^{N}I_{x,k}^{2}\right)\left(\sum_{k=1}^{N}I_{y,k}I_{k}\right)}{\det(H)}$$

(4) $$H=\left[\begin{array}{cc} \sum_{k=1}^{N}I_{x,k}^{2} & \sum_{k=1}^{N}I_{x,k}I_{y,k}\\ \sum_{k=1}^{N}I_{x,k}I_{y,k} & \sum_{k=1}^{N}I_{y,k}^{2} \end{array}\right],\det(H)=\left(\begin{array}{c} \sum_{k=1}^{N}I_{x,k}^{2} \end{array}\right)\left(\begin{array}{c} \sum_{k=1}^{N}I_{y,k}^{2} \end{array}\right)-{\left(\begin{array}{c} \sum_{k=1}^{N}I_{x,k}I_{y,k} \end{array}\right)}^{2}$$

where $I_{x},\;I_{y}$ represent the gradient of neighboring pixels, H denotes the Hessian matrix, and N is the total number of pixels in this window. After detecting corners, the external parameters of a single image are solved. For each image, the OpenCV library is used to solve the rotation R and translation t of the calibration board relative to the camera through 3D-2D point pairs. The principle is shown in Equation (5):

(5) $$\min_{R,t}\sum_{i}\|p_{i}-\pi (K\cdot (R\cdot P_{i}+t)){\|}^{2}$$

which denotes the 3D coordinates of the calibration-board corners in the world frame, and $p_{i}$ denotes the detected 2D image coordinates. π is the projection function, i.e., the complete camera model that maps a 3D point onto the 2D image plane, rather than the mathematical constant π. In particular, Equations (2) and (3) refine the corner locations from pixel-level estimates to sub-pixel accuracy to reduce noise. Equation (5) then uses the refined high-precision corners to estimate the camera intrinsic and extrinsic parameters. The objective of Equations (2)–(5) is to minimize the overall reprojection error of all corners, thereby improving calibration accuracy.

After obtaining the camera intrinsics and extrinsics, a global optimization is performed to jointly refine the intrinsics, extrinsics, and distortion coefficients. Specifically, the OpenCV library is used to formulate the reprojection error of all corners as the objective function and to optimize the parameters accordingly. The core of the calibration optimization is the minimization of the reprojection error over all detected corners.

(6) $$E=\sum_{i=1}^{N}\sum_{j=1}^{M}\|p_{ij}-{\hat{p}}_{ij}(K,dist,R_{j},t_{j}){\|}^{2}$$

where N denotes the number of calibration images, M denotes the number of corner points per image, ${\hat{p}}_{ij}$ is the predicted coordinate calculated via the projection formula, K is the optimized internal reference matrix, and dist is the distortion coefficient. The external parameters for each image are $R_{j}$ and $t_{j}$. E represents the reprojection error, measured in pixels squared. This error is the cumulative sum of all corner point positional errors across all images. Upon completion of the camera calibration experiment, the system ultimately calculates the reprojection error E for each corresponding camera.

After completing the experiment, the reprojection error E for the left camera, right camera, and RGB camera must be individually verified to determine the validity of the calibration results. The reprojection error threshold for the left and right cameras is set to 1.111, while the threshold for the RGB camera is set to 3.0. If the reprojection error of each camera does not exceed its corresponding threshold, the calibration process is deemed to meet accuracy requirements and the calibration results are considered valid.

### 3.2. Improved 3D Reconstruction Method for ORB-SLAM3

**Figure 2:**
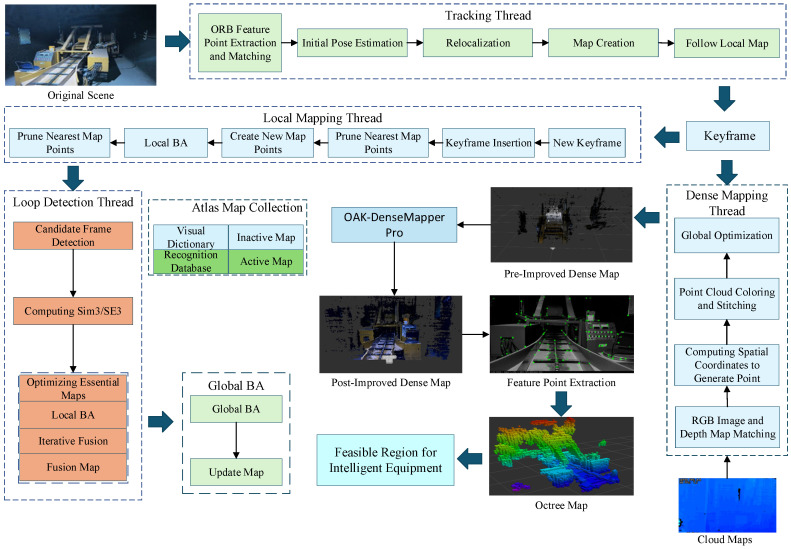
Schematic Diagram of the Improved ORB-SLAM3 Algorithm.

To address the challenge of constructing feasible domains for intelligent mining equipment operating in complex, enclosed, and dynamic environments, this paper proposes a spatial feasible domain representation and construction method that integrates RGB-D depth cameras with an enhanced ORB-SLAM3 algorithm. The main framework of the proposed algorithm is illustrated in Figure 2.

The ORB-SLAM3 system generates dense maps from data captured by an RGB-D camera calibrated using the ChArUco calibration board. The dense map point cloud generated by the original ORB-SLAM3 system is sparse. In coal mine tunnels, intelligent equipment utilizes octrees constructed from dense maps to represent navigable areas. While octrees built from sparse point clouds can detect the approximate locations of obstacles, they fail to identify small obstacles. Moreover, sparse point clouds lead to rough or discontinuous reconstructed surfaces, making octrees unsuitable for high-fidelity modeling. To address this, integrating the OAK-DenseMapper Pro system into the original algorithm enhances dense map mapping performance and improves dense point cloud detail. This approach effectively refines dense map construction.

### 3.3. Coupled Dense Map Construction Method for the OAK-DenseMapper Pro System

#### 3.3.1. Introduction to the OAK-DenseMapper Pro System

**Figure 3:**
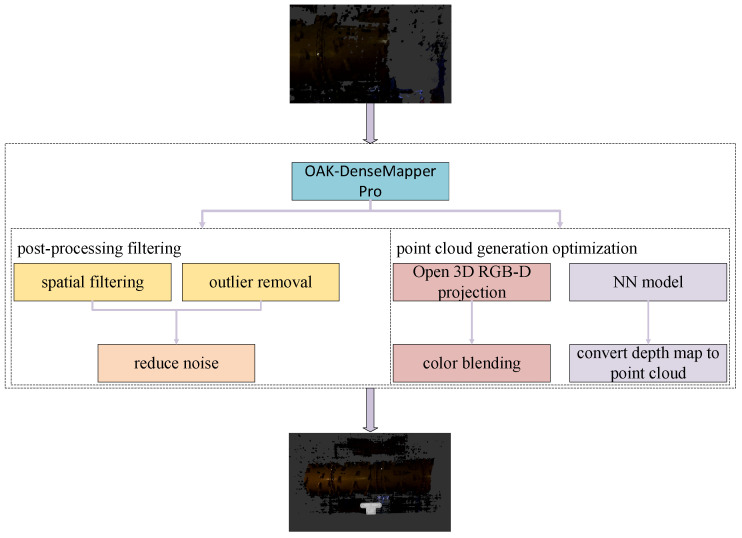
Framework Structure of the OAK-DenseMapper Pro System.

The OAK-DenseMapper Pro system is a real-time RGB-D point cloud generation and interactive visualization system. It enhances dense map construction by optimizing point cloud generation based on mathematical projection models and applying post-processing filtering. The overall system architecture is illustrated in Figure 3.

#### 3.3.2. Point Cloud Data Optimization Method Based on Mathematical Projection Models

The camera captures images through left and right monocular cameras and generates depth maps using the parallax method. The underlying mathematical principle is:

(7) $$Z=\frac{f\cdot B}{d}$$

z represents the depth value, f denotes the focal length, B indicates the baseline distance, and d signifies the parallax value. After generating the depth map, the next step is to convert it into point cloud information. First, define the PyTorch model in depth_to_3d.py [29] to generate a 3D point cloud by combining the depth map with the precomputed XYZ grid. Next, export the model to ONNX format and convert it to a format supported by the camera.

Pixel coordinates are transformed into normalized coordinates via the camera’s intrinsic matrix. The mathematical principles underlying point cloud conversion are illustrated in Equations (8)–(10):

(8) $$x=\frac{u-c_{x}}{f_{x}},\;\;\;\;y=\frac{v-c_{y}}{f_{y}}$$

Combined with the depth value  z (in millimeters), the three-dimensional coordinates are obtained:

(9) X=x⋅z,    Y=y⋅z,    Z=z

This is achieved by element-wise multiplication of the precomputed XYZ grid with the depth map. Subsequently, a point cloud is generated by multiplying the depth value Z with the normalized coordinates on a point-by-point basis:

(10) P=[x⋅Z,y⋅Z,Z]

After point cloud transformation, the original camera coordinate system must be converted to the world coordinate system. The point cloud orientation is adjusted using rotation matrix $R_{camera\to world}$ to align with the standard world coordinate system where the *Y*-axis points downward and the *Z*-axis points forward.

(11) $$R_{camera\to world}=\left[\begin{array}{ccc} 1 & 0 & 0\\ 0 & -1 & 0\\ 0 & 0 & -1 \end{array}\right]$$

The PyTorch model defined in this paper is not a traditional deep learning model, but rather a lightweight projection model whose core function is to rapidly convert depth maps into 3D point clouds. This model is fundamentally a mathematical transformer based on geometric rules, designed to efficiently execute fixed algorithms. In contrast, traditional deep learning models are data-driven function approximators that learn complex input-to-output mappings through training [30]. The fundamental difference lies in whether they rely on data-driven parameter optimization and possess generalization capabilities. Specific distinctions are outlined in Table 1.

The point cloud data optimization method based on mathematical projection models fully leverages hardware acceleration capabilities to achieve efficient point cloud generation with minimal computational overhead, providing a real-time, reliable 3D data source for dense mapping. To present the point cloud data optimization method based on the mathematical projection model in an intuitive manner, the pseudo-code of this method is shown as Algorithm 1:
**Algorithm 1** Point Cloud Data Optimization Method Based on Mathematical Projection Models** Input:** Depth image D ∈ ℝ^{H × W}, Camera intrinsic parameters K = {$f_{x}$, $f_{y}$, $c_{x}$, $c_{y}$} ** Output:** Dense 3D point cloud P 1: **Initialize empty** point cloud P ←∅ 2: **for** each pixel (u, v) ∈ D **do** 3:  z ← D(u, v) 4:  **if** z **is** invalid **then** 5:    **continue** 6:  **end if** 7:  x ← ($u-c_{x}$)·z/$f_{x}$ 8:  y ← ($v-c_{y}$)·z/$f_{y}$ 9:  p ← (x, y, z) 10:   **Add** p **to** P 11:   **end for** 12: **return** P

Algorithm 1 describes how to generate a dense point cloud based on the refined depth map through a mathematical projection model. For each valid pixel in the depth map, first, the pixel coordinates are normalized using the camera intrinsic parameters to obtain the ray direction in the camera coordinate system; then, the ray is back-projected to the three-dimensional space using the corresponding depth value to generate the three-dimensional points in the camera coordinate system. To ensure geometric consistency within the system, the generated points are further transformed to the unified world coordinate system through the predefined rotation matrix. By back-projecting pixel by pixel and excluding invalid depth points, this algorithm can efficiently reconstruct a dense and geometrically consistent three-dimensional point cloud from the depth data.

#### 3.3.3. Point Cloud Data Optimization Method Based on Stereo Matching and Multi-Stage Filtering

Generating dense point clouds relies on the precise alignment of high-precision depth maps with RGB images, followed by post-processing optimization. This section enhances point cloud quality through four key techniques: stereo matching optimization, depth map filtering, coordinate alignment, and point cloud post-processing.

Stereo matching optimization calculates disparity maps from left and right images, improving depth resolution and robustness through sub-pixel interpolation, left-right consistency checks, and extended disparity range. The sub-pixel accuracy formula is:

(12) $$d_{subpixel}=d_{integer}+\frac{\Delta d}{32}$$

where Δd represents the difference between adjacent disparity values, and 32 denotes the number of sub-pixels. Subsequently, left-right consistency is verified, and erroneous matching points within occluded regions are eliminated as shown in Equation (13).

(13) $$Valid(d)=\left\{\begin{array}{ll} 1 & if/d_{left}(x,y)-d_{right}(x-d,y)/<range\\ 0 & otherwise \end{array}\right.$$

Depth map filtering is a process that applies a series of algorithms to raw depth maps to remove noise, fill holes, and enhance data quality. The primary goal of depth map filtering is to provide accurate and reliable depth information for subsequent point cloud generation and 3D reconstruction. The filtering techniques applied in this paper include median filtering, spatial-temporal filtering, and threshold filtering. These filtering methods collectively enhance depth map quality through noise suppression, mismatch removal, hole filling, and data truncation, laying the foundation for generating high-precision dense point clouds.

The purpose of median filtering is to remove salt-and-pepper noise, while temporal filtering employs multi-frame weighted averaging to reduce jitter, as illustrated by Equation (14):

(14) $$D_{t}=\alpha D_{t-1}+(1-\alpha )D_{\mathrm{current}}$$

The core objective of temporal filtering is to address missing values in depth maps caused by occlusion, weak texture, or noise through local pixel operations within a single frame depth map. Its principle is illustrated in Equation (15):

(15) $$D_{filled}(x,y)=\frac{1}{N}\sum_{i,j\in neighborhood}D\left(x+i,y+j\right)$$

Coordinate alignment refers to projecting 3D points from a depth map onto the RGB image plane using camera calibration parameters, ensuring color and geometry are aligned. This process utilizes the extrinsic matrix:

(16) $$T_{depth2rgb}=[R|t]$$

from the depth camera to the RGB camera:

(17) $$P_{rgb}=T_{depth2rgb}\cdot P_{depth}$$

The projection formula is:

(18) $$\left[\begin{array}{c} u\\ v\\ 1 \end{array}\right]=K_{rgb}\cdot \frac{P_{rgb}}{Z_{rgb}}$$

where $K_{rgb}$ is the intrinsic matrix of the RGB camera.

Server-side optimization achieves high-quality dense point cloud generation through stereo matching optimization, depth map filtering, coordinate alignment, and point cloud post-processing. Its core lies in depth map preprocessing and geometric transformations, while Open3D’s efficient API further streamlines the point cloud generation workflow. To present the point cloud data optimization method based on stereo matching and multi-stage filtering in an intuitive manner, the pseudo-code of this method is shown in Algorithm 2:
**Algorithm 2** Point Cloud Data Optimization Method Based on Stereo Matching and Multi-stage Filtering **Input:** Depth image D, RGB image I, Depth camera intrinsics $k_{d}$, RGB camera intrinsics $k_{rgb}$, Extrinsic transformation $T_{\left\{d\to rgb\right\}}=(R,t)$
 **Output:** Dense colored point cloud $p_{c}$ 1: **Initialize empty** point cloud $p_{c}\leftarrow \emptyset$ 2: **for** each pixel (u,v) ∈ D
**do** 3:  z ← D(u,v) 4:  **if** z is invalid **then** 5:    **continue** 6:  **end if**     //Back-project depth pixel to 3D (depth camera frame) 7:  $x_{d}$ ← (u − $c_{x}^{d}$)·z/$f_{x}^{d}$ 8:  $y_{d}$← (v − $c_{y}^{d}$)·z/$f_{y}^{d}$ 9:  $p_{d}$ ← ($x_{d}$, $y_{d}$, z)       //Transform to RGB camera frame 10:   $p_{rgb}$ ← $R\cdot p_{d}$ + t       //Project to RGB image plane 11:   $u_{rgb}$ ←$f_{x}^{rgb}$·$p_{rgb}^{x}$/$p_{rgb}^{z}$ + $c_{x}^{rgb}$ 12:   $v_{rgb}$ ← $f_{y}^{rgb}$·$p_{rgb}^{y}$/$p_{rgb}^{z}$ + $c_{y}^{rgb}$ 13:   **if** ($u_{rgb}$, $v_{rgb}$) lies inside image bounds **then** 14:     c ← I($u_{rgb}$, $v_{rgb}$) 15:     **Add** ($p_{rgb}$, c) to $p_{c}$
 16:   **end if** **17**: **end for**
 **18**: **return**
$p_{c}$


Algorithm 2 constructs a dense point cloud generation and optimization framework at the system level, driven by stereo vision geometric constraints. Through multi-stage consistency constraints and coordinate transformations, pixel-level depth observations are mapped into a three-dimensional geometric representation in a unified coordinate system, thereby supporting subsequent dense mapping and spatial modeling processes. It should be noted that the theoretical analysis adopted in this paper is based on the geometric characteristics of voxel downsampling. The maximum spatial error introduced is limited by half of the diagonal length of the voxel, thus allowing for the constraint of spatial fidelity loss without relying on additional geometric truth data.

### 3.4. Construction of the Feasible Domain Voxel Space for Intelligent Equipment

Dense point cloud maps contain three-dimensional information, but their massive data volume demands substantial storage space and computational resources. In dynamic environments, octree maps offer superior update efficiency and real-time performance compared to dense maps, significantly reducing map reconstruction time and securing critical time windows for safe obstacle avoidance decisions. Therefore, octree maps are employed to represent the feasible voxel space for intelligent mining equipment.

The improved dense map generation for octree maps is based on an occupancy probability model. Let b ∈ R denote the probability log value and a be the probability between 0 and 1. The occupancy probability for each voxel between them is updated via a logit transformation.

(19) $$b=logit(a)=log\left(\frac{a}{1-a}\right)$$

The inverse transformation calculation yields the final probability as:

(20) $$a=\frac{\exp(b)}{\exp(b)+1}$$

This process dynamically models the occupancy probability of obstacles and supports incremental updates. When a node continuously observes occupancy, the b-value increases. When observing vacancy, the b-value decreases. Mathematically, let node be, and observation data be. Then, from time to time, the probability log-value of the node is $L\left(n|z_{1:t}\right)$, and at time  t+1 is:

(21) $$L(n|z_{1:t+1})=L(n|z_{1:t-1})+L(n|z_{t})$$

If we express the above equation in probabilistic terms, then:

(22) $$p(n|z_{1:t})={\left[1+\frac{1-p(n|z_{t})}{p(n|z_{t})}\cdot \frac{1-p(n|z_{1:t-1})}{p(n|z_{1:t-1})}\cdot \frac{p(n)}{1-p(n)}\right]}^{-1}$$

With the logarithmic probability, the entire octree map can be updated based on RGB-D data. Suppose a pixel with depth d is observed in the RGB-D image. This indicates that an occluding object was detected at the spatial point corresponding to that depth value, and no objects exist along the line segment from the camera’s optical center to that point. Using this information, the octree map can be updated to accommodate moving structures.

The updated octree recursively divides 3D space into voxels, with each node representing a cubic region. The root node covers the entire 3D scene, while child nodes are obtained through binary recursive division of 3D space. Given a 3D spatial region:

(23) $$\Omega \subset R^{3}$$

In Equations (19)–(23), voxel occupancy states are modeled and updated using a log-odds probability representation. The occupancy probability a∈(0,1) is mapped into the log-odds domain, which converts probabilistic updates into additive updates. This formulation effectively avoids numerical instability that may arise from repeated multiplications/divisions in probability space, and it also facilitates the fusion of observations across multiple time steps.

In dynamic environments, uncertainty in voxel occupancy primarily originates from depth measurement noise of the RGB-D sensor, occlusion effects, and errors introduced when continuous space is discretized into voxels. To characterize and propagate this uncertainty, we perform Bayesian updates of voxel occupancy probabilities in the log-odds domain. When the sensor detects an obstacle at a voxel location, a positive log-odds increment is assigned to that voxel; when a sensor ray passes through a voxel without observing an obstacle, a negative log-odds increment is applied. With the accumulation of multiple independent observations, measurement uncertainty naturally propagates and is gradually reflected in the voxel occupancy probabilities.

To prevent a single noisy observation or long-term accumulated errors from excessively influencing voxel states, the occupancy probability is constrained within a predefined interval $\left[P_{min},P_{max}\right]$ during the update process. This avoids probability saturation toward 0 or 1 and improves the numerical stability and robustness of map updates. In addition, an occupied threshold $P_{occ}=0.7$ and a free-space threshold $P_{free}=0.3$ are introduced for voxel state classification: voxels with occupancy probability higher than $P_{occ}$ are classified as occupied, those lower than $P_{free}$ are classified as free space, and voxels between the two thresholds are treated as unknown.

The choice of $P_{occ}=0.7$ and $P_{free}=0.3$ balances observation noise, environmental dynamics, and mapping stability, while explicitly preserving uncertain regions to prevent frequent oscillations between occupied and free states. Retaining this unknown state preserves uncertainty and helps reduce misclassification under degraded sensing conditions, while suppressing the propagation of errors in space.

Although an explicit analytical error-propagation model is not derived in this work, the log-odds Bayesian update scheme, probability bounding, and threshold-based voxel state classification together provide an effective mechanism to model and implicitly propagate occupancy uncertainty. The experimental results reported later indicate that the proposed approach can produce stable and consistent 3D feasible-space representations in complex dynamic environments.

Its initial bounding box is:

(24) $$B_{0}=[x_{\min},x_{\max}]\times [y_{\min},y_{\max}]\times [z_{\min},z_{\max}]$$

The octree partitioning rule divides the cubic region of each node by splitting it at the midpoint of its three-dimensional coordinates:

(25) $$x_{c}=\frac{x_{min}+x_{max}}{2},\;y_{c}=\frac{y_{min}+y_{max}}{2},\;z_{c}=\frac{z_{min}+z_{max}}{2}$$

The eight regions obtained after division are:

(26) $$B_{ijk}=\left[x_{min}^{\left(i\right)},x_{max}^{\left(i\right)}\right]\times \left[y_{min}^{\left(j\right)},y_{max}^{\left(j\right)}\right]\times \left[z_{min}^{\left(k\right)},z_{max}^{\left(k\right)}\right],i,j,k\in \{0,1\}$$

where x can be expressed as:

(27) $${x_{min}}^{\left(0\right)}=x_{min},\;\;\;\;{x_{max}}^{\left(0\right)}=x_{c},\;\;\;\;{x_{min}}^{\left(1\right)}=x_{c},\;\;\;\;{x_{max}}^{\left(1\right)}=x_{max}$$

Similarly, y and z can be derived. Let F denote the feasible region as the union of all octahedron containing map points. From Equations (26) and (27), we know that the spatial representation of the octree is:

(28) $$F=\underset{n\in N}{\cup }B_{n}$$

where n denotes the node number in the octree, N represents the node set of the octree, and each $B_{n}$ corresponds to the spatial extent associated with a particular node in the octree.

During the map construction process in ORB-SLAM3, the three-dimensional space is first bounded by an enclosing cube. Based on this, a recursive division is performed using an octree structure. Each division uses the midpoints of the cube along the xxx, yyy, and zzz axes as boundaries, splitting it into eight sub-cubes. As the division progresses, the system gradually forms a hierarchical spatial grid structure. Since map points are derived from triangulated feature points, their distribution determines the scope of the feasible region. Consequently, only sub-cubes containing at least one map point need be retained, while empty sub-cubes are discarded. By merging all sub-cubes containing map points, an octree-based representation of the feasible region is obtained. This approach ensures sparse spatial representation while significantly improving query and management efficiency.

### 3.5. Complexity and Space Fidelity Analysis and Discussion of Environmental Robustness

The mapping framework proposed in this paper adopts an octree-based occupancy representation to efficiently describe large-scale tunnel environments. In what follows, we analyze the computational complexity of the occupancy update process from the perspectives of sensor observations and map scale.

For each incoming RGB-D frame, ray casting is performed for all valid depth measurements. Let R denote the number of rays per frame. This value is determined by the sensor resolution and can be treated as a constant when the camera configuration is fixed. During propagation, each ray intersects a finite number of voxels, denoted by *k*, whose upper bound is jointly determined by the sensor’s maximum range and the preset voxel resolution. Therefore, k can be regarded as a local constant independent of the global environment scale. Each voxel state update requires accessing and modifying the corresponding node in the octree. This involves traversing from the root to the leaf node, and the associated cost is proportional to the tree depth d. In practice, the octree depth typically grows logarithmically with the number of allocated voxels N, i.e., d=O(logN), where N denotes the number of active voxel nodes in the current map. Combining these factors, the per-frame runtime complexity of the proposed method can be expressed as:

(29) O(R⋅k⋅d)

This cost is dominated by local ray traversal and voxel access along an octree path of logarithmic depth. Although the total number of allocated voxels increases as tunnel length grows, the per-frame computation only updates voxels within the current observation range. As a result, the computational burden is largely insensitive to the overall map scale. This theoretical result is consistent with the runtime scaling experiments reported in Section 3.5.

When converting a dense point cloud into an octree voxel representation, some loss of spatial detail is unavoidable. To quantify this effect, we analyze spatial fidelity from voxel geometry. Let the voxel resolution be *r*. After voxelization, the theoretical upper bound of the spatial deviation is limited by half of the voxel diagonal:

(30) $$\varepsilon_{\max}=\frac{\sqrt{3}}{2}r$$

In our experiments, the voxel resolution is set to 0.05 m, corresponding to a maximum spatial error of approximately 0.043 m. This error magnitude is considerably smaller than the typical structural dimensions and safety-clearance requirements of underground intelligent equipment, and thus does not lead to a substantive impact on feasible-space determination or obstacle-avoidance decisions.

The above analysis indicates that, with a reasonable voxel resolution, the geometric error introduced by voxelization remains controllable and does not materially affect feasible-space judgment. Nevertheless, in practical underground coal-mine settings, mapping accuracy is influenced not only by voxelization but also by environmental factors such as dust, high humidity, and complex lighting.

The underground environment of a coal mine is usually characterized by high dust concentration, high humidity, and a large number of metal components. These factors have a significant impact on visual perception and depth measurement. Dust and uneven lighting can lead to unstable features and increased depth noise. The humid environment and mirror reflection of metal surfaces may cause depth abnormalities or incorrect matching. To address these issues, this paper improves the reliability of camera calibration and depth estimation through an RGB-D perception method based on ChArUco calibration. In the dense reconstruction process, multi-stage depth filtering is introduced to suppress abnormal measurements. At the same time, the probability occupancy modeling method based on the octree is used to accumulate and update the results of multiple frames, and the uncertain areas are explicitly retained. This reduces the influence of instantaneous dust occlusion, humid reflection, and metal reflection on the determination of the feasible region, enhancing the robustness of the system in harsh underground environments.

## 4. Experimental Results and Analysis

The hardware and software configuration for this experiment’s runtime environment was Ubuntu 20.04-ROS1, with an NVIDIA RTX 4060 GPU and AMD Ryzen 7 CPU.

### 4.1. Camera Calibration Experiment Using the ChArUco Calibration Board

**Figure 4:**
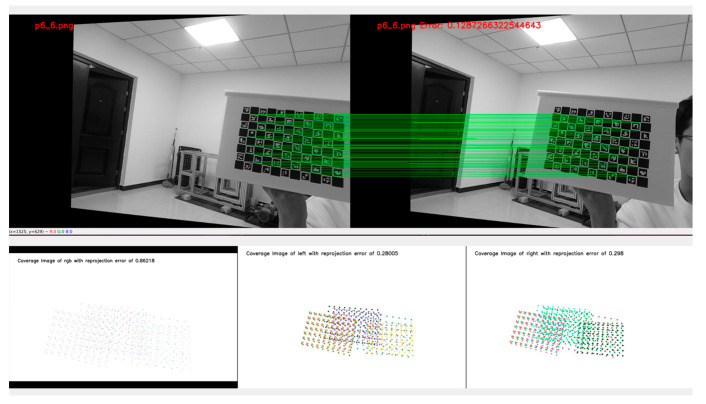
Camera Calibration Experiment.

The calibration experiment first captures 13 sets of images, followed by error analysis. If the E-values of both the left and right cameras and the RGB camera are below the error threshold, calibration is successful. To mitigate experimental variability and errors, four trials were conducted, with the lowest overall error selected as the final result. The calibration experiment is illustrated in Figure 4.

The experimental results are shown in Table 2, where $E_{1}$ denotes the reprojection error of each camera in Experiment 1. A comprehensive comparison of the four experiments reveals that in Experiment 4, both the RGB camera and the left-right cameras meet the error threshold requirements, with the lowest overall error levels. Therefore, all subsequent experiments in this paper are based on the data from $E_{4}$.

### 4.2. Feature Point Extraction and Depth Mapping Experiments

**Figure 5:**
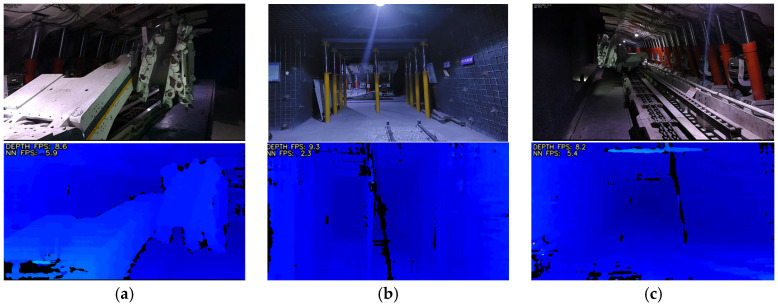
Depth Point Cloud Information Map for Different Scenarios in Underground Coal Mines. (**a**) Cutting head for fully mechanized mining face; (**b**) Airway Door-Type Support Area; (**c**) Hydraulic Support for Fully Mechanized Mining Face.

To evaluate the system’s environmental perception capabilities and validate the algorithm’s mapping performance in complex scenarios, subsequent experiments were conducted in the main laboratory of the coal mine. Within the laboratory, depth cameras captured images of intelligent equipment and the underground environment to generate depth point clouds. Figure 5 displays the original images of detection objects in the first row and their corresponding depth maps in the second row.

Each pixel in the three depth maps shown in Figure 5 records distance information from the camera to the target surface, representing a two-dimensional form with sparse depth features. Depth maps serve as a crucial input for dense map reconstruction. After registration and fusion of multiple depth maps acquired from different viewpoints, a dense 3D map with continuous surface structure can be generated. The objective of this experiment is to provide foundational data and technical groundwork for subsequent dense map construction and precision enhancement.

### 4.3. Comparative Experiment of Dense Mapping with Improved Algorithm

The improved ORB-SLAM3 algorithm in this paper demonstrates significant optimization in both overall mapping quality and local mapping details, with substantially reduced point cloud redundancy.

**Figure 6:**
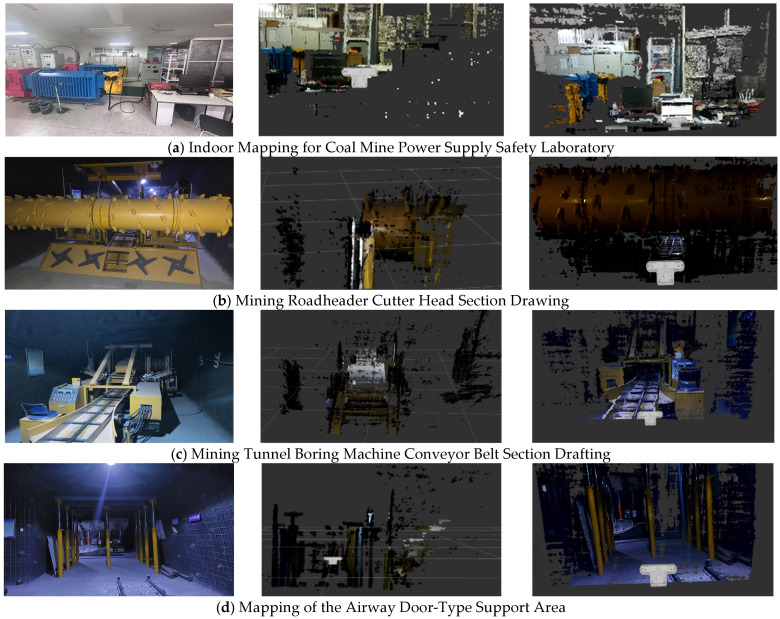
Comparison of Mapping Performance Across Multiple Scenarios Before and After Algorithm Improvement.

Figure 6 shows a comparison of dense map construction before and after the algorithm improvement. Figure 6a shows the original scene (1), the dense map constructed by the original ORB-SLAM3 system (2), and the dense map constructed by the improved ORB-SLAM3 system (3). The main laboratory of the coal mine has insufficient light and poor visibility. In order to construct the described object more clearly, the image of the original scene (1) in the six scenes was processed with supplementary lighting during the photography. However, the lighting effect in the actual mapping process of (2) and (3) is far inferior to that of (1).

Figure 6a demonstrates how the algorithm improves dense map completeness in well-lit laboratory environments, enhancing the detail of described objects. Figure 6b,c shows that even under extremely poor lighting conditions in coal mine tunnels, the improved algorithm can still construct clear and complete dense maps of described objects. Figure 6d illustrates enhanced dense map detail and more complete point cloud images under uneven lighting conditions. The improved ORB-SLAM3 algorithm substantially addresses the issue of incomplete mapping caused by sparse point clouds in dense mapping scenarios. Following the algorithmic enhancements, the number of point clouds in the same scene increased by 38%, as shown in Table 3. Overall, the improved ORB-SLAM3 significantly enhances the effectiveness of dense mapping.

### 4.4. Algorithm Trajectory Error Evaluation Analysis

**Figure 7:**
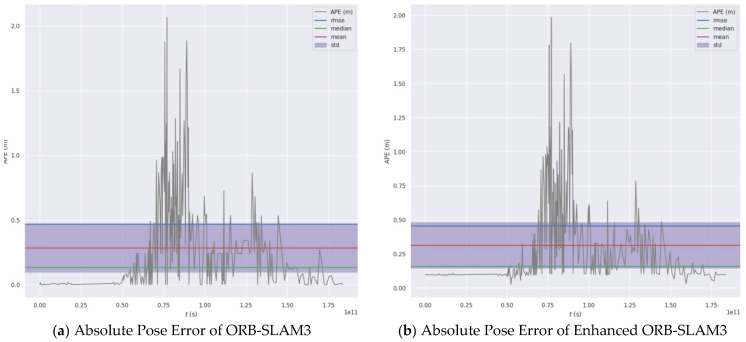
Absolute Pose Error Before and After Algorithm Enhancement.

To ensure statistical reliability of the trajectory evaluation, we conducted repeated runs on the TUM dataset. A representative indoor sequence (fr1) was selected, and under identical sequences and identical parameter configurations, ORB-SLAM3 and the proposed method were each executed multiple times independently. Absolute Pose Error (APE) was used as the evaluation metric and computed with the evo tool. Before evaluation, all estimated trajectories were time-synchronized with the ground-truth trajectories and aligned using a rigid transformation. For each sequence, the mean and standard deviation (mean ± std) over multiple runs were reported to reflect both stability and dispersion. To reduce the influence of randomness, algorithm parameters were fixed and differences introduced by random initialization were constrained under controlled conditions. All reported trajectory-error improvements are based on statistical averages rather than single-run results. The results are summarized in Figure 7, where RMSE denotes root-mean-square error, Median denotes the median value, Mean denotes the arithmetic mean, and Std denotes the standard deviation.

In the comparative experiment, we introduced the SGDO-SLAM algorithm [31] and the VGS-SLAM algorithm [32] for comparison. After comparing the data of SGDO-SLAM, VGS-SLAM, ORB-SLAM3 and the improved algorithm, we obtained Table 4. Among them, compared with ORB-SLAM3, the root mean square error (RMSE) of the improved algorithm was reduced by 7.7%, the arithmetic mean error (Mean) was reduced by 7.1%, the median error (Median) was reduced by 10%, and the standard deviation (Std) was reduced by 8.7%. In all indicators, the improved ORB-SLAM3 algorithm is better than the VGS-SLAM algorithm. The experiment shows that in complex scenarios, the positioning accuracy of the algorithm in this paper is better than that of the original algorithm and the VGS-SLAM algorithm. The algorithm in this paper can better adapt to the complex and closed environment of coal mines and realize the construction of the feasible domain of intelligent equipment in coal mines.

### 4.5. Experiment on Voxel-Based Feasible Domain Construction for Intelligent Equipment in Underground Coal Mines

Octree maps exhibit significantly superior updatability and optimizability compared to dense point cloud maps, enabling the construction of higher-precision feasible domain voxel spaces.

**Figure 8:**
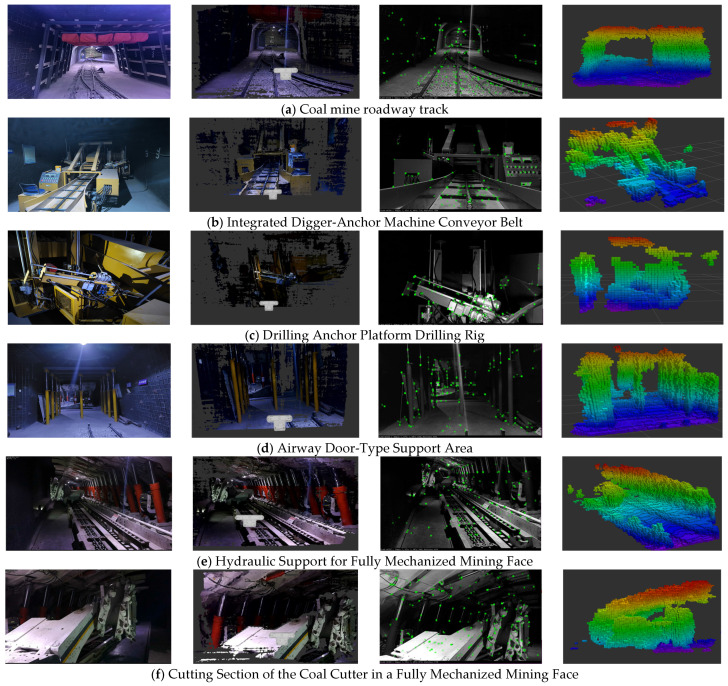
Spatial Construction of Feasibility Volumes for Intelligent Equipment in Different Areas of Underground Coal Mines.

The octree-occupied map uses a fixed voxel side length r = 0.05 m for voxelization and incremental updates. No additional multi-resolution resampling is performed in this paper. The maximum number of feature points per frame of the ORB feature extractor is ORBextractor.nFeatures = 2000. The four columns in Figure 8 show: the original scene (left 1), the dense map constructed by the improved ORB-SLAM3 algorithm (left 2), feature point extraction of the target object (left 3), and the octree map constructed by the improved ORB-SLAM3 algorithm (left 4).

**Figure 9:**
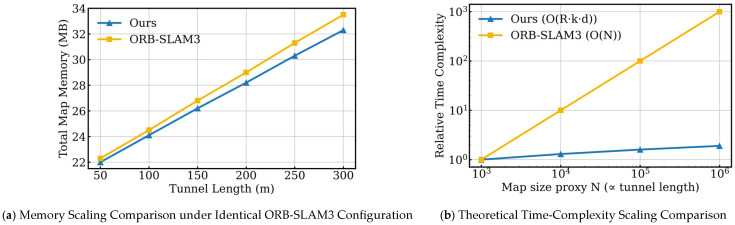
Comparison of computational scalability between ORB-SLAM3 and the method presented in this paper.

Figure 8 shows the construction of the feasible domain voxel space of intelligent equipment in different areas of a coal mine roadway. Figure 8b–f shows the construction of the intelligent equipment itself and its feasible domain voxel space. In the octree map, the area not occupied by the colored squares is represented as the feasible domain of the intelligent equipment. Figure 9a compares the map memory scaling behavior of ORB-SLAM3 and the proposed method under identical configuration settings as the tunnel length increases. Both approaches exhibit an approximately linear increase in memory usage, which is consistent with the theoretical analysis presented earlier that, under fixed tunnel cross-sections and voxel resolutions, the map size grows linearly with tunnel length. However, compared with ORB-SLAM3, the proposed method maintains a consistently lower memory footprint across all tunnel lengths, and the gap between the two curves gradually widens as the tunnel length increases. Figure 9b shows the theoretical time-complexity scaling curves, where the map size N is plotted on the horizontal axis and the relative computational complexity on the vertical axis, using a log–log scale. As illustrated, the complexity curve of ORB-SLAM3 increases approximately linearly with *N*(O(N)), reflecting the fact that the computational cost associated with dense point-cloud maintenance, map data accumulation, and related processing grows significantly as the environment scale expands. In contrast, the proposed method relies on octree-based occupancy updates; when the sensor resolution and voxel resolution are fixed, the per-frame computational complexity is mainly determined by the octree traversal depth. Consequently, the growth trend of the proposed method, characterized by O(R⋅k⋅d), is markedly more gradual. This indicates that, as tunnel length and map scale increase, the proposed method exhibits weaker dependence on the global environment size, making it more suitable for long-distance, fixed or semi-fixed underground spaces such as coal-mine tunnels, where long-term stable operation and real-time feasible-space construction are required.

As shown in Table 5, converting the dense map generated by the improved ORB-SLAM3 algorithm into an octree map takes approximately 0.75 s. Under identical conditions, the memory space occupied by the octree map constructed by the improved algorithm exhibits an exponential decrease compared to that of the dense map it generates. This achieves a shift from megabytes to kilobytes. As shown in Table 6, the converted map information occupies only about 0.5% of the original memory, significantly reducing memory consumption. The octree map stores occupancy information describing the map, enabling better real-time scalability and low-cost fulfillment of the construction of feasible domains for the working space of intelligent equipment in coal mines. It also provides a digital space for subsequent trajectory planning of intelligent equipment.

## 5. Conclusions

This paper proposes a method for constructing the dynamic feasible region of intelligent equipment in coal mines based on depth cameras and an improved ORB-SLAM3. Through calibration experiments using the ChArUco calibration board, low-error, high-precision camera calibration was achieved. Through The OAK-DenseMapper Pro System That Integrates a Mathematical Projection Model and Optimizes RGB Camera Data Acquisition—the approach enhances dense map construction and improves detail in octree map generation. The improved algorithm better adapts to complex underground environments, enabling the representation and construction of intelligent equipment’s voxel feasible domain within intricate mining conditions. This paper provides a digital space for subsequent trajectory planning of intelligent equipment combined with trajectory planning algorithms.

Although RTAB-Map and TSDF-based mapping methods are widely adopted in mobile robotics, they typically assume that the sensor moves with the platform and rely on active exploration and global map optimization. Under our fixed-viewpoint, space-constrained feasible-space modeling setting, it is difficult to integrate and compare these methods under fully matched assumptions and parameter configurations. Therefore, we do not include them as direct baselines in this study, and systematic comparisons will be pursued in future work.

The proposed system is currently in the development and validation stage. After implementation and debugging on the ROS1 platform, experiments were conducted in a highly realistic coal-mine laboratory environment to evaluate the feasibility and stability of the algorithm under the target operational scenario. System-level testing under extreme conditions in real mines will be carried out in future work once on-site deployment conditions become available.

Key conclusions are as follows:

1. This work establishes an ORB-SLAM3–based framework for 3D feasible-domain modeling in underground coal mines. By leveraging the high-accuracy pose estimation provided by ORB-SLAM3 and integrating RGB-D depth sensing with dense 3D reconstruction, the proposed system enables coordinated localization and feasible-space representation, providing a systematic solution for digital modeling of complex operational spaces.
2. This paper proposes a dense reconstruction method based on ChArUco calibration and multi-stage depth optimization, aiming to improve the mapping quality in complex environments. By combining high-precision ChArUco calibration with mathematical projection models, stereo matching, and multi-stage depth filtering, it effectively addresses the issues of sparse dense point clouds and missing details under weak texture and low illumination conditions, providing a reliable geometric foundation for the subsequent construction of the feasible region’s voxelization.
3. The dense point cloud is converted into a probabilistic octree occupancy map, where voxel updates are performed only for observed space. We further model the computational complexity of the feasible-space construction process and derive a per-frame time complexity of O(R⋅k⋅d), where *R* is the number of depth rays, k is the number of voxels traversed per ray, and d is the octree depth. In addition, we show that memory usage grows approximately linearly with the spatial scale. Together, theoretical analysis and experimental results confirm the scalability and real-time capability of the proposed method.

## Figures and Tables

**Table 1:** Comparison of Mathematical Projection Models and Traditional Deep Learning Models.

Aspects	Mathematics Projection Model	Traditional Deep Learning
Model Structure	Mathematical Projection	Multi-layer neural network
Training Requirements	Direct mathematical definition without training	Requires a large amount of labeled data for training
Training Resource Requirements	Minimal	Extensive
Applications	Real-time Point Cloud Generation	Classification, detection, etc.

**Table 2:** Camera Error Analysis.

Camera	$E_{1}$	$E_{2}$	$E_{3}$	$E_{4}$	Error Threshold
RGB Cam.	1.0	2.9	1.3	0.9	3.0
L. Cam.	0.356	0.303	0.332	0.280	1.111
R. Cam.	0.332	0.295	0.272	0.298	1.111
Average	0.344	0.299	0.302	0.289	1.111

**Table 3:** Comparison of Point Cloud Quantities in Dense Maps Before and After Algorithm Improvement.

Dense Map	ORB-SLAM3	Enhanced ORB-SLAM3	Enhance Ratio
a	271,963	376,618	38%
b	173,247	238,524	38%
c	142,921	198,625	39%
d	186,529	254,405	36%

**Table 4:** Error Analysis Before and After Algorithm Improvement.

Indicator	SGDO-SLAM	VGS-SLAM	ORB-SLAM3	Ours	Reduction Ratio
Rmse	0.957 m	0.049 m	0.052 m	0.048 m	7.7%
Mean	0.815 m	0.029 m	0.028 m	0.026 m	7.1%
Median	0.759 m	0.010 m	0.010 m	0.009 m	10%
Std	0.503 m	0.024 m	0.023 m	0.021 m	8.7%

**Table 5:** Comparison of Mapping Time Before and After Algorithm Improvement.

No.	ORB-SLAM3 Map Creation Time	Algorithm Construction Time in This Paper	Reduction Ratio
a	0.88 s	0.76 s	13.6%
b	0.84 s	0.75 s	15.5%
c	0.83 s	0.71 s	14.5%
d	0.90 s	0.76 s	15.6%
e	0.89 s	0.77 s	13.5%
f	0.85 s	0.75 s	11.8%

**Table 6:** Comparison of Memory Requirements for Dense Maps and Feasible Region Voxel Spaces.

No.	Feasible Region F	Original Image Memory	Point Cloud Memory	Octree Memory
a	$\underset{n\in N_{a}}{\cup }B_{n}^{a}$	677 KB	5.1 MB	25.5 KB
b	$\underset{n\in N_{b}}{\cup }B_{n}^{b}$	514 KB	4.3 MB	21.7 KB
c	$\underset{n\in N_{c}}{\cup }B_{n}^{c}$	749 KB	4.2 MB	20.7 KB
d	$\underset{n\in N_{d}}{\cup }B_{n}^{d}$	623 KB	5.5 MB	27.2 KB
e	$\underset{n\in N_{e}}{\cup }B_{n}^{e}$	683 KB	5.9 MB	28.4 KB
f	$\underset{n\in N_{f}}{\cup }B_{n}^{f}$	550 KB	6.1 MB	30.1 KB

## Data Availability

The data used to support the findings of this study are not publicly available due to privacy and confidentiality considerations, but are available from the corresponding author upon reasonable request.
